# Isolation and characterization of a spontaneously immortalized bovine retinal pigmented epithelial cell line

**DOI:** 10.1186/1471-2121-10-33

**Published:** 2009-05-04

**Authors:** Thomas E Liggett, T Daniel Griffiths, Elizabeth R Gaillard

**Affiliations:** 1 Department of Biological Sciences, Northern Illinois University, DeKalb, IL, USA; 2 Current address: Department of Neurological Sciences, Rush University Medical Center, Chicago, IL, USA; 3 Department of Biological Sciences, Marquette University, Milwaukee, WI, USA; 4 Department of Chemistry and Biochemistry, Northern Illinois University, DeKalb, IL, USA

## Abstract

**Background:**

The Retinal Pigmented Epithelium (RPE) is juxtaposed with the photoreceptor outer segments of the eye. The proximity of the photoreceptor cells is a prerequisite for their survival, as they depend on the RPE to remove the outer segments and are also influenced by RPE cell paracrine factors. RPE cell death can cause a progressive loss of photoreceptor function, which can diminish vision and, over time, blindness ensues. Degeneration of the retina has been shown to induce a variety of retinopathies, such as Stargardt's disease, Cone-Rod Dystrophy (CRD), Retinitis Pigmentosa (RP), Fundus Flavimaculatus (FFM), Best's disease and Age-related Macular Degeneration (AMD). We have cultured primary bovine RPE cells to gain a further understanding of the mechanisms of RPE cell death. One of the cultures, named tRPE, surpassed senescence and was further characterized to determine its viability as a model for retinal diseases.

**Results:**

The tRPE cell line has been passaged up to 150 population doublings and was shown to be morphologically similar to primary cells. They have been characterized to be of RPE origin by reverse transcriptase PCR and immunocytochemistry using the RPE-specific genes *RPE65 *and *CRALBP *and RPE-specific proteins RPE65 and Bestrophin. The tRPE cells are also immunoreactive to vimentin, cytokeratin and zonula occludens-1 antibodies. Chromosome analysis indicates a normal diploid number. The tRPE cells do not grow in suspension or in soft agar. After ^3^H thymidine incorporation, the cells do not appear to divide appreciably after confluency.

**Conclusion:**

The tRPE cells are immortal, but still exhibit contact inhibition, serum dependence, monolayer growth and secrete an extra-cellular matrix. They retain the *in-vivo *morphology, gene expression and cell polarity. Additionally, the cells endocytose exogenous melanin, A2E and purified lipofuscin granules. This cell line may be a useful *in-vitro *research model for retinal maculopathies.

## Background

The retinal pigmented epithelium (RPE) is a post-mitotic monolayer of neuroepithelial-derived cells [1,2]. The role of retinal pigment epithelial cells in vision disorders, such as Stargardt's disease, Best's macular dystrophy, retinitis pigmentosa, cone-rod dystrophy and age-related macular degeneration (AMD) is considered to be extremely important [3-5]. Due to the diverse number of functions of RPE cells, the degree of these debilitating diseases resulting from improper functioning can cause a cascade of events that result in RPE and photoreceptor cell death, which causes permanent vision loss [6-8]. The greatest vision loss occurs in the macular region due to its high concentration of photoreceptor and RPE cells and its role in central vision. Around the age of 30, the RPE begins progressive age-related changes. These include an increase in lipids and residual bodies in the basal lamina, an increase in basal cytoplasmic infoldings, which can be caused by an accumulation of residual bodies, the appearance of lipid material between the RPE and Bruch's membrane and an accumulation of the age-related pigment lipofuscin [9-16].

In order to understand the various roles of RPE cells, investigators have grown these cells *in-vitro*. Unfortunately, there are many obstacles in the culture of primary RPE cells. Because these cells are post-mitotic *in-vivo*, they do not proliferate well in culture. Those that do grow will begin to lose many of the characteristics of the *in-vivo *state within the first few passages. These changes include depigmentation, reduced or altered gene expression, changes in morphology, loss of cell junctions and an alteration in metabolic function [17-21]. Initially, RPE research overcame this problem of dedifferentiation by isolating primary cells from a large number of animals, but with varying degrees of genetics. The process was not only time intensive, but also expensive. By varying the growth conditions, such as the addition of all-trans retinoic acid, ascorbic acid and other compounds, it was possible for RPE cells to regain some of the characteristics observed with *in-vivo *cells [18,22-27]. This provided researchers with the ability to carry out experiments on cultures with the same genetic make-up. Although this has advantages over the constant dissection of tissue, eventually the cells will senesce. Alternatively, there have been some RPE cultures that have been induced to be immortal by transfection with viruses, such as SV40 or telomerase [28-30].

We have isolated and characterized a spontaneously immortalized RPE cell line from calf eyes, called tRPE. Sub-culturing of individual primary colonies has produced a cell line that has undergone over 150 population doublings, but still exhibits many of the *in-vivo *characteristics. They grow as a monolayer, are contact inhibited, produce an extra-cellular matrix, and can be maintained in culture for over a year. No transformed foci were detected, but individual cells did form colonies when grown under low-density conditions. The cell line exhibits RPE-specific gene expression as determined by reverse transcriptase PCR and immunocytochemistry.

## Results

### RPE morphology

**Figure 1:**
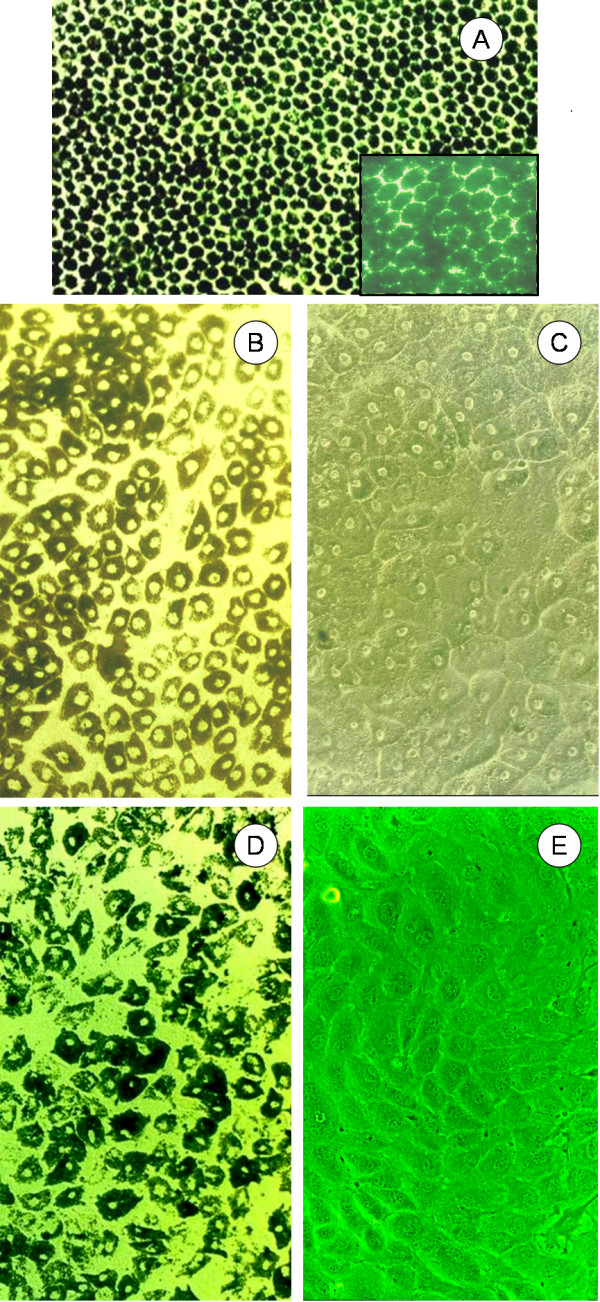
**Morphological comparison between in vivo RPE, freshly excised RPE and tRPE cells**. **A) **Melanotic RPE *in vivo *morphology. **B) **Photograph of a confluent passage 1 primary melanotic RPE culture with the inset as a close-up. **C) **Photograph of passage 5 primary RPE cells displaying a polygonial morphology. **D) **Photograph of passage 102 tRPE cells which were pigmented by feeding them exogenous melanosomes. **E) **Photograph of confluent passage 102 tRPE cells grown on bovine corneal endothelial extracellular matrix (BCE-ECM).

Primary RPE sheets were isolated using a gelatin isolation protocol as an indicator of the *in vivo *morphology. The morphology of these cells was used as a comparison for the optimal outcome for the RPE cell tissue cultures. The primary RPE cells were hexagonal in shape and densely pigmented throughout (Figure 1A) as observed by phase contrast microscopy. It should be noted that even the center of the cell contained pigment covering the nucleus.

Primary cells that were trypsinized had attached to tissue culture plastic dishes generally within one week. Proliferation of the cells generally began one to two weeks after attachment. The cells increased in size by approximately four-fold and the shape of each individual cell became polygonial (Figure 1B). The nucleus was visible with melanin localized in a perinuclear arrangement. When the cells were at passage 5 (Figure 1C), they were totally devoid of pigment, but still retained the epithelioid morphology. The nucleus was indented and was visible by phase contrast microscopy. Cultures were maintained to approximately 35 passages before they senesced.

A new RPE cell line was generated from calf eyes that were dissected and grown on tissue culture plates. Seven primary RPE cell explants were isolated by cloning rings and grown under low-density conditions. Colonies were re-isolated to generate pure cultures. The cells were allowed to become confluent and then passaged. Of these clones, only one produced a cell line that surpassed 50 population doublings and eventually was passaged to over 150 population doublings. This cell line was designated as tRPE. They have the ability to endocytose melanin (Figure 1D) after feeding the cells isolated and purified melanosomes, and yielded a similar morphology as the pigmented primary cells. The control cells 3T3, HeLa and UV61 did not take up the melanosomes (data not shown).

When grown on top of BCE-ECM, the unpigmented tRPE cells (Figure 1E) more closely resembled the primary cell morphology (Figure 1C). They were flat, polygonal, and they did not grow as dense as tRPE cells grown on Matrigel or tissue grade plastic.

### Reverse transcriptase PCR

RT-PCR was performed to detect the expression of four genes. Since other cell types can contaminate cultures from crude dissection, the RPE-characteristic genes, RPE65 and CRALBP, were used as markers for the identification of RPE cell origin. Additionally, both RPE65 and CRALBP expression have been shown to be diminished *in-vitro *[17,31], so they were also used as markers for RPE differentiation. Expression of β-actin was used as a quantitative marker, since it is a ubiquitous housekeeping gene. Epithelial cells do not normally express vimentin, but epithelial cells grown in tissue culture and in ARMD patients will express this gene [19,32,33].

**Figure 2:**
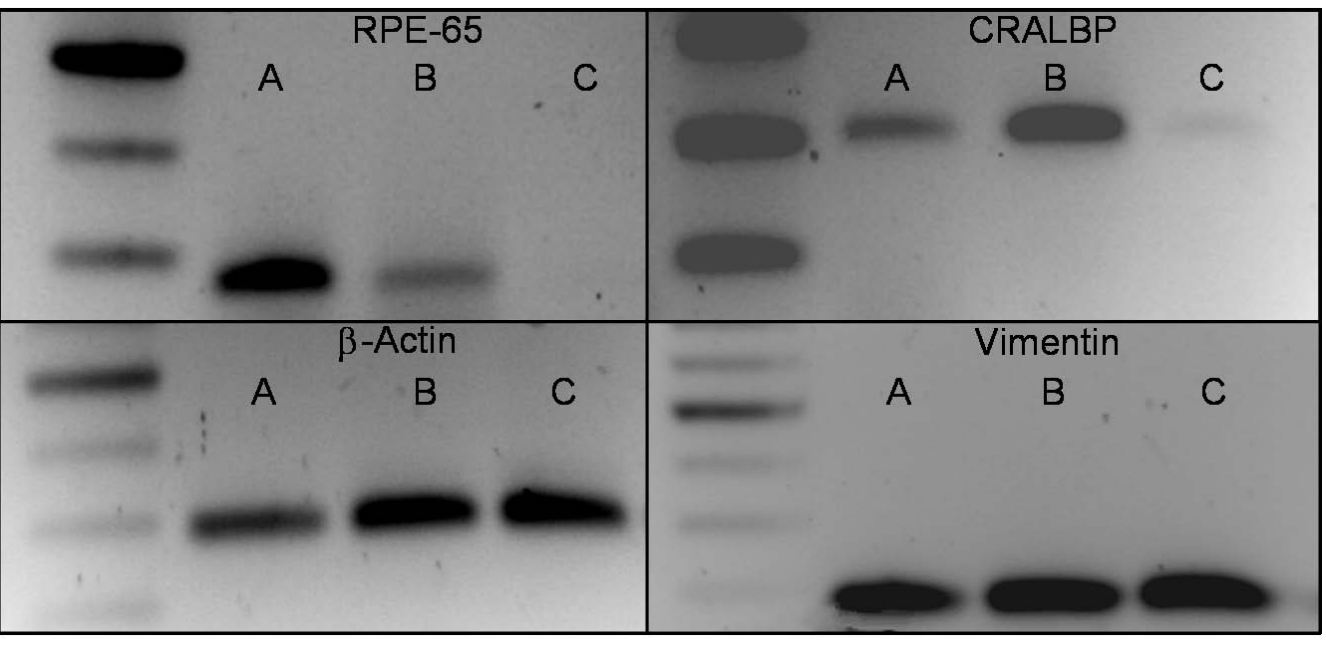
**Agarose gel electrophoresis of the reverse transcriptase PCR reactions of different cell cultures under various conditions**. Molecular weight markers are located on left side with the respective sizes on the right. For each panel, the respective gene is labeled above each agarose gel. **A) **RPE cells extracted from the eyecup; **B) **Passage 100 tRPE cells grown on BCE-ECM for two months in conditioned media; **C) **Passage 100 tRPE cells grown on plastic for two months in normal DMEM.

Figure 2 shows the gel electrophoresis of the RT-PCR products. The primary cells express RPE65, CRALBP, β-actin and vimentin. The tRPE cells do not express RPE65 and only minimally express CRALBP when grown on plastic as compared to the primary cells. Upon culturing on bovine corneal endothelial extra cellular matrix (BCE-ECM), the expression of CRALBP is more prominent and the expression of RPE65 appears. All of the cultured cells express vimentin with varying degrees. The control cell line CHO-AA8 does not express RPE65 or CRALBP, but does express β-actin (Data not shown).

### Immunocytochemistry

**Figure 3:**
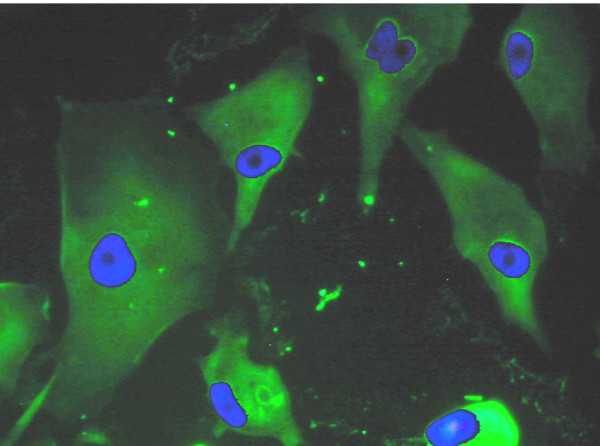
**Immunocytochemistry of tRPE cells expressing RPE-65**. RPE-65 immunocytochemistry of tRPE cells grown on BCE-ECM using conditioned media. The green photograph was taken through the FITC filter cube showing the localization of RPE-65, and overlayed was the blue photograph taken through the DAPI filter cube showing the nuclear counterstain Hoechst 33258.

Expression of two RPE-specific genes was analyzed. RPE65 was immunolocalized in tRPE cells (Figure 3) throughout the cells. The green fluorescence from the FITC conjugated antibody was seen throughout the cells, but they had a mottled appearance. Also, there was higher emission intensity towards the edges of the cells.

**Figure 4:**
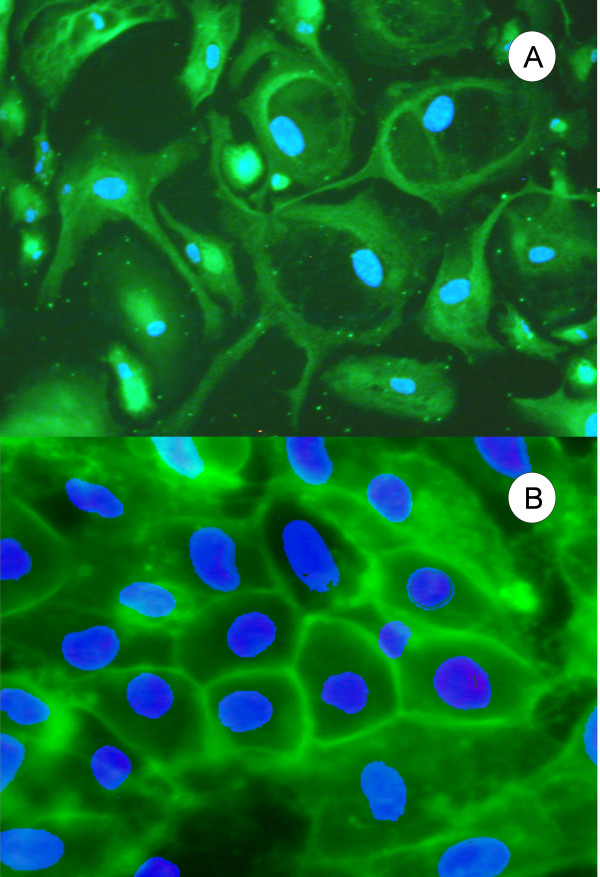
**Immunocytochemistry showing the polarity of tRPE cells**. **A) **Bestrophin immunocytochemistry of tRPE cells grown on BCE-ECM using conditioned media. The green photograph was taken through the FITC filter cube showing the localization of bestrophin and the photograph was overlayed with the blue photograph taken through the DAPI filter cube showing the nuclear counterstain Hoechst 33258; **B) **Zonula Occludens-1 (ZO-1) immunocytochemistry of tRPE cells grown on BCE-ECM using conditioned media. The green photograph was taken through the FITC filter cube showing the localization of ZO-1 around the periphery of the cells and the photograph was overlayed with a blue photograph taken through the DAPI filter cube showing the nuclear counterstain Hoechst 33258.

The other RPE-specific protein analyzed was bestrophin (Figure 4A). There was heavy localization of the protein seen on the plasma membrane towards the periphery. The protein was also seen as less intense on the interior, but the intensity could have been diminished because it was not within the same focal plane. The cells nuclei were counterstained with Hoechst 33258. The expression of zonula occludens-1 (ZO-1) was determined as a marker for the formation of epithelial tight junctions. Figure 4B shows that the cellular location of the protein was in a hexagonal shape on the periphery of the cells.

**Figure 5:**
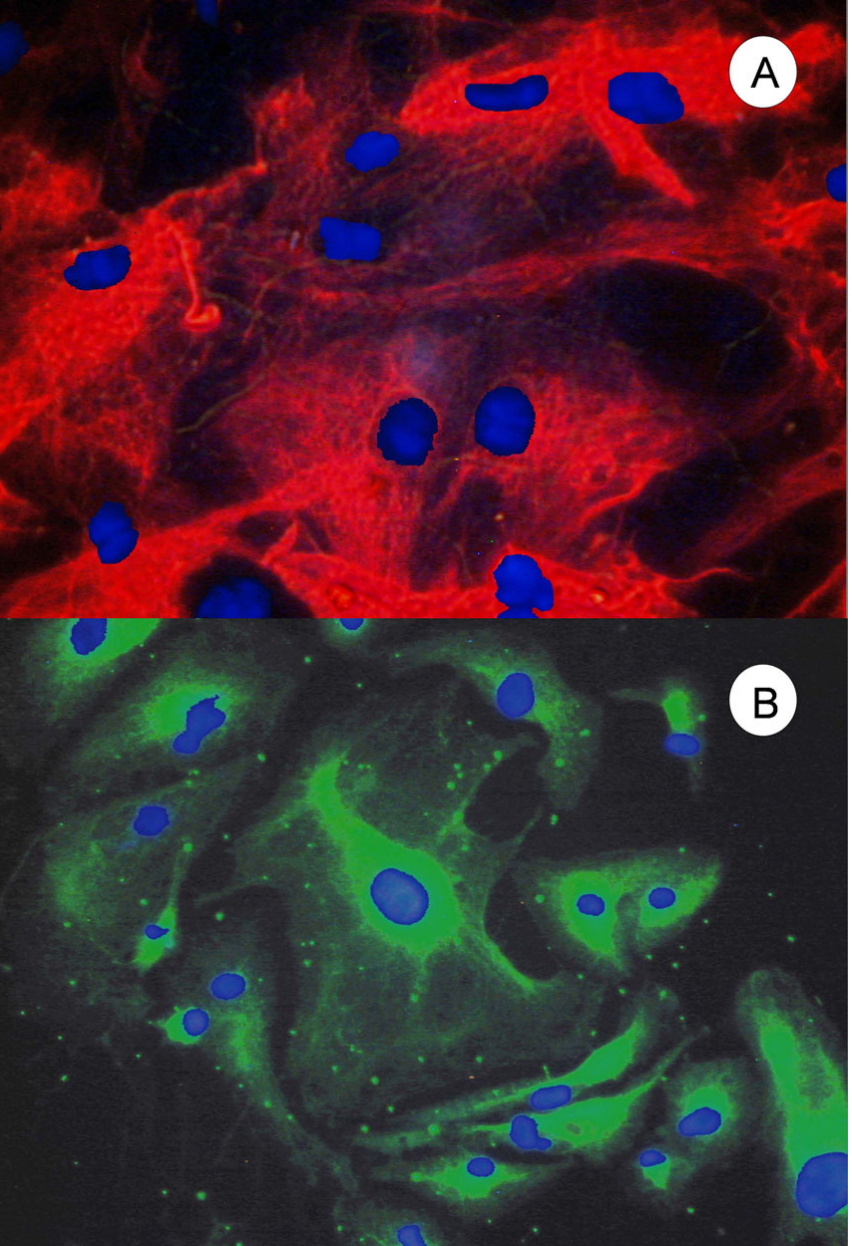
**Immunocytochemistry of tRPE cells expressing cytoskeletal proteins**. **A) **Vimentin immunocytochemistry of tRPE cells grown on BCE-ECM using conditioned media. The red photograph was taken through the TRITC filter cube showing the finger-like projections of vimentin, which was overlayed with a blue photograph taken through the DAPI filter cube showing the nuclear counterstain Hoechst 33258. **B) **Pan-cytokeratin immunocytochemistry of tRPE cells grown on BCE-ECM using conditioned media. The green photograph was taken through the FITC filter cube showing the localization of the cytokeratin, and overlayed with a blue photograph taken through the DAPI filter cube showing the nuclear counterstain Hoechst 33258.

Cytoskeletal proteins were also analyzed. Figure 5A illustrates a tRPE culture that showed positive immunoreactivity to the anti-vimentin antibody. The red fluorescence shows a radiating pattern from the nucleus that was Hoechst counterstained in blue. Most of the cells stained intensely for vimentin. Additionally, the expression of pan-cytokeratin by the tRPE cells is seen in Figure 5B. The cells had a more intense fluorescence that was localized around the nucleus and fingerlike extensions that radiated outwards.

### Transformation tests

**Figure 6:**
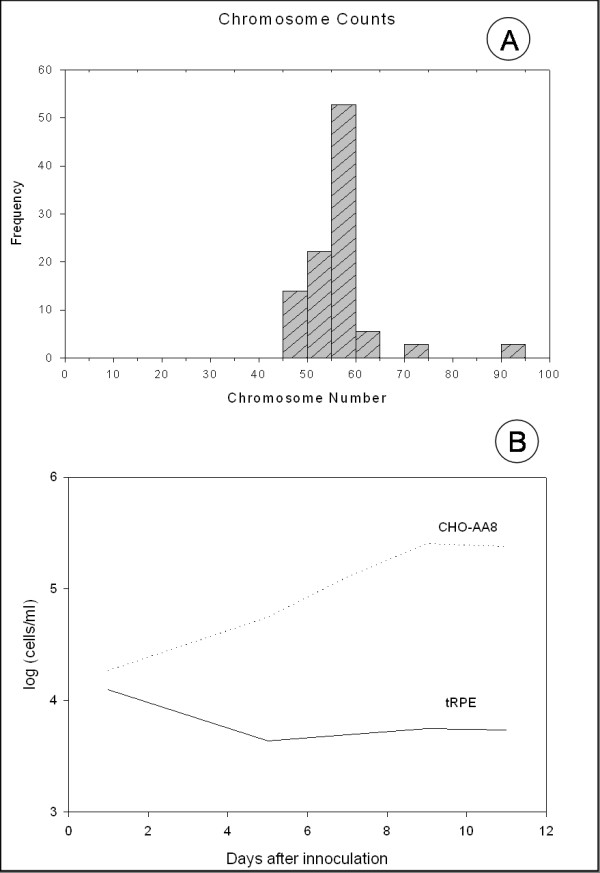
**Graphical analysis of transformation experiments using tRPE cultures**. **A) **Frequency of chromosome counts of passage 102 tRPE cells (n = 63); **B) **Growth curves of passage 102 tRPE cells compared to CHO cells grown in suspension cultures.

Chromosome counts of the tRPE cells varied only slightly from the normal diploid number of 60 [34], which is indicative of non-transformed cells (Figure 6A). All of the chromosomes were telocentric except for two, and many of the chromosomes were small. It was believed that counts that were less than 60 chromosomes arose from the loss of chromosomes during the fixation process when hypotonic cell solutions were dropped onto the slides.

Growth of cells in a roller drum apparatus inhibits cells from attaching to the sides. Epithelial cells require some substrate, whether an ECM or tissue culture plastic, to attach in order to survive [24]. Transformed cells can become anchorage-independent. The graph in Figure 6B compares the tRPE cell line to CHO-AA8. Clearly, the tRPE cells did not divide in culture. Their cell number rapidly declined after initial incubation. The CHO-AA8 cells continued to grow until they reached a steady state.

**Figure 7:**
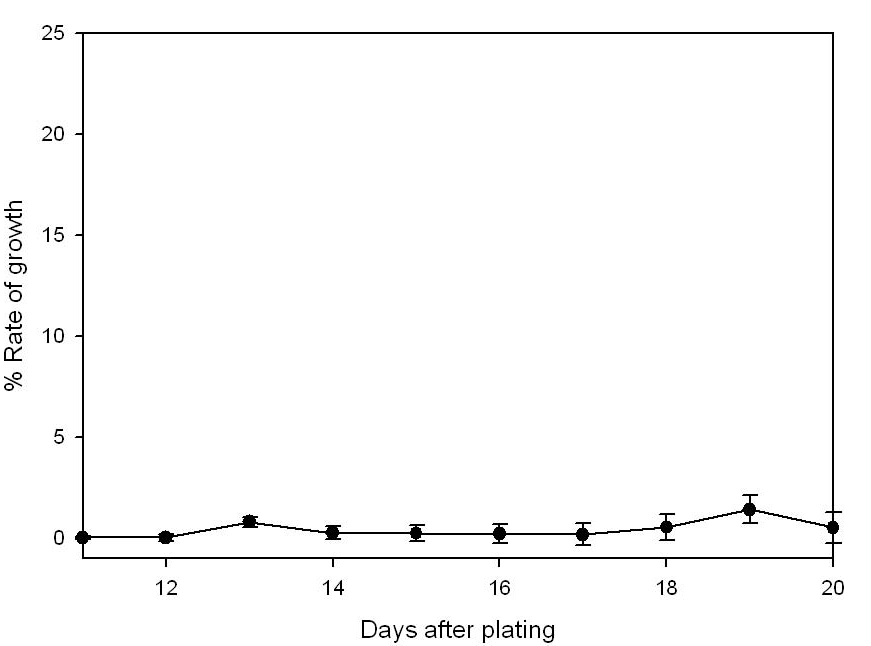
**^3^Thymidine emulsion autoradiography of passage 102 tRPE cultures**. The percentage of cells incorporating ^3^thymidine at various times after plating shows that the cells do not divide appreciably after confluency (note the y-axis has been truncated for clarity).

The ^3^H thymidine emulsion autoradiography assay can be used to determine the total percentage of cells that are dividing at a given time. Since transformed cells generally have a faster rate of proliferation than untransformed cells, the assay is also an indicator of whether the cells are transformed. The cells that take up the thymidine will generate a high density of silver grains around the nucleus of the cell after exposure to the emulsion. The number of cells that had incorporated silver grains was compared to the total number of cells in a given area. The graph in Figure 7 shows that a maximum of 1.5% of the total cells had divided, but most time points generated values less than 1%.

## Discussion

Many mouse cell lines have undergone spontaneous immortalization or transformation in tissue culture [35-39], but in general, cells from most of the higher species do not undergo spontaneous immortalization or transformation [40]. The problem is further compounded in RPE research because the cells are post-mitotic *in-vivo *and therefore must first re-enter the cell cycle. Also, removal of primary RPE cells from Bruch's membrane causes a majority of cells to die in culture. Apart from the commonly utilized ARPE-19, there are few accounts of spontaneously immortalized RPE cell lines such as D407, RPE-J and BPEI-1 [41,42]. Additionally, it is common practice for primary cells to be transformed by viruses, such as SV40 or HPV, or other genetic manipulation such as activating a telomerase gene [28-30,43,44]

While our cell line, tRPE, still shows many of the *in-vivo *characteristics, including expression of RPE-specific genes, further experiments would have to be performed to determine whether the spontaneous immortalization that occurred had changed the expression of other genes.

Primary RPE cell isolation can often become contaminated with choroidal melanocytes or fibroblasts. The melanocytes are derived from neural crest embryonic tissue whereas RPE are optic cup derived [1,45]. These melanocytes can often be confused with the pigmented RPE, but melanocytes are difficult to culture in normal unsupplemented media. TPA, bFGF, phorbol esters, or concurrent growth with keratinocytes are required to grow the melanocytes [46,47].

The tRPE cell line had similar morphological characteristics as the passaged primary cells. Although the tRPE cells were never observed to have the hexagonal morphology as seen *in-vivo *cells, they were polygonal. Growth of RPE cells on BCE-ECM and Matrigel has been established and used by many investigators [24,48-51]. Other researchers have shown that primary RPE and other RPE cell lines grow better on BCE-ECM than Matrigel [49,50]. Because ECM is integral in the differentiation of epithelial cells, both types of ECM were used to determine the optimal matrix material.

Morphologically, tRPE cells and primary cells grown on BCE-ECM were epithelioid. When Matrigel was used, the cells were elongated and disorganized, and did not seem to inhibit the proliferation of RPE cells (data not shown). Based upon morphological characteristics, BCE-ECM seemed to be a better substrate for tRPE cells.

It is important to have the cells melanized in order to regain an *in-vivo *like state. There are many publications that state melanin is a protective agent to light mediated damage and also acts as a free radical quencher [52]. Remelanization has been attempted by feeding the cells α-MSH and thymidine dimmers, and irradiation with UV (data not shown). Boulton and Marshall [53], and Denton et al. [44] have shown that directly feeding purified melanosomes to the cells causes the cells to endocytose exogenous melanin. In our research, we have found that immortalized RPE cells will take up melanosomes granules when fed in culture, but a control cell line, HeLa, does not. The exogenously melanated tRPE cells were similar in morphology to primary cells as seen in Figure 1. Although speculative, the melanin granules may cause the tRPE cells to re-differentiate, but this needs further study.

Addition of growth factors was attempted as a mechanism to increase the differentiation of RPE cells. Although serum-free media has been established for RPE cultures, the cost and time for preparing it prohibited its use [51,54]. Some of the ingredients were supplemented into the DMEM media as a cost-effective approach. The supplementation of the media along with growth on BCE-ECM was a requirement to fully differentiate the tRPE cells in order to determine if they produced RPE-specific proteins.

The tRPE cell line was characterized to establish that they are indeed RPE origin. Four genes were used as identifying markers performed by RT-PCR. *RPE65 *and *CRALBP *are RPE-specific genes [17,55-61]. *RPE65 *expression is rapidly lost in cells that have been cultured without any external signals [17,31,62], and growth of RPE on BCE-ECM has been shown to produce an increase in *CRALBP *expression [24]. We have seen that the tRPE cells will express both *RPE65 *and *CRALBP *when grown on BCE-ECM with supplemented media. This indicates that the tRPE cells may have the ability to re-differentiate when grown under these conditions. As expected, the *RPE65 *expression was lost in the tRPE cells cultured on tissue grade plastic, and *CRALBP *expression was diminished.

Vimentin functions as an intracellular scaffold, and it is expressed in cultured RPE cells and primary cells from ARMD patients [19,63]. We show that both tRPE and primary RPE cells express the gene. Finally, *β-actin *is a housekeeping gene and was used as a measure to quantitate the relative levels of expression. We believe that the RT-PCR data support that the clones derived from the primary explant are of RPE origin.

To further expand the evidence that the cells are of RPE origin and retain the *in-vivo *characteristics, immunocytochemistry was performed. Reverse transcriptase PCR was already performed, but it does not show that the cell will produce protein. There are post-transcriptional regulations that can inhibit the translation of the protein. Multiple groups have confirmed that cultured RPE cells will express ZO-1, RPE65, and CRALBP under specific conditions [8,24,64,65]. Expression was determined by immunocytochemistry to show that the cells do produce proteins specific to RPE cells and some that are characteristic of monolayer epithelial cells.

Because Nicoletti et al. have shown that the RPE65 mRNA has AU rich elements on the 3' UTR that will allow down-regulation of the RPE65 protein, [57], the presence of the protein needed to be established. Only after the cells have redifferentiated when grown on an extracellular matrix, is the expression seen again [17,31]. Ma et al. have shown that a Sf9 cell line transfected with a plasmid-containing recombinant human RPE65 will have a distribution throughout the cell when detected with an anti-RPE65 antibody [57]. Figure 3 shows that the tRPE cells produce RPE65. The green fluorescence is seen throughout the cells, but has a mottled appearance and higher intensity toward the edges of the cells, which may be indicative of protein on the membrane and in the cytosol.

Vimentin is usually used as a marker for the determination of cell types of mesenchymal origin [66-68], while cytokeratins are found in epithelial tissue almost exclusively [69]. Both of these proteins are frequently co-expressed with in RPE cultures. Rapidly dividing cells show cytokeratin diffusely, while quiescent cells will show a strongly staining perinuclear cage-like structure surrounded by profuse finger-like projections extending out from the nucleus [70]. Immunocytochemistry showed that most of the cells were positive for vimentin and cytokeratin, but not all. The tRPE cells were reactive against antibodies to both proteins in a typical pattern of the cytoskelatal network found in RPE cultured cells, which further indicates that the cells are of RPE origin.

Long culture conditions are needed to form adherins junctions in RPE cells [64,65], which forms an adhesion belt around each cell [69]. Zonula Occludens-1 (ZO-1), also known as tight junction protein (TJP1), is utilized in adherins junctions for cell polarization, transport and cell-to-cell signaling [71-73]. ZO-1 immunofluorescence shows that the tRPE cells form a nearly hexagonal structure on the periphery of the cells, as previously seen in Figure 1a. This indicates that the tRPE cells form adherins junctions and are a polarized epithelial monolayer, as expected of differentiated RPE.

Bestrophin is heavily detected in cells of epithelial and endothelial origin because of the requirement of these cells in transport of nutrients through the cell [74,75]. Marmorstein et al. have shown bestrophin to localize in the basolateral side of RPE cells to control the transport of chloride and water between the RPE and the choriocapillaris [74]. The observed expression of this protein indicates the tRPE cells have a polarized morphology and are a monolayer of epithelial cells.

Initially, the tRPE cells were thought to be spontaneously immortalized because they had no foci formation after the cells became confluent. Generally, as cells increase in passage number, they have the ability to become transformed due to mutations accumulating in their DNA. Specific tests were done to establish that the tRPE cells were not transformed even at population doubling 102. Additionally, the experiments also show that the cells still retained specific characteristics of a differentiated state.

The tRPE cells are spontaneously immortalized after being passaged up to 150 population doublings. Growth beyond 100 population doublings is considered immortal [76]. The cells did not have the ability to grow in the lack of serum or under low serum conditions and did not form foci (data not shown), which are two characteristics of untransformed cells.

Bovine cells have a normal diploid chromosome number equal to 60 [34]. All of the autosomes are telocentric except the X and Y chromosomes, which are metacentric [77]. Transformed cells have been shown to be aneuploid or heteroploid [78]. Most of the chromosomes that were counted were within the 55–60 range, with many at 60 chromosomes.

Growth studies of cells in suspension or soft agar were performed to determine whether the cells were anchorage-independent and exhibited the loss of contact inhibition [79]. The tRPE cell numbers dramatically decreased in suspension at the beginning of the experiment, indicating they require a substrate to attach. The tRPE cells were unable to grow in soft agar, while the control CHO AA8 cells were able (data not shown).

Pulse labeling was performed to confirm that the cultures stopped or slowed down proliferation after confluence, a process called density-dependent inhibition of cell growth [80]. The number of dividing cells was never greater than 1.5%, with most times being less than 1%. Comparatively, actively dividing tRPE cells were expected to have a rate of labeling around 25%, and transformed cells could show up to 50% of cells labeled with ^3^H-thymidine. The tRPE cells did not exhibit significant cell division at confluency. Also, the tRPE cells remained as a confluent culture for one year and still were viable, whereas primary cells are difficult to keep alive for any extended period of time.

## Conclusion

The RPE-characteristic genes and proteins RPE65, CRALBP, and bestrophin were expressed in the tRPE cell line using the combination of BCE-ECM and conditioned media. The tRPE cell line is an improvement over primary cell lines, because it is immortal, and therefore, does not senesce. The tRPE cells exhibit characteristics of non-transformed cells such as contact inhibition, serum dependence and monolayer growth, but the cells have reached 150 population doublings. From the results, it appears that the tRPE cells produce a stable RPE cell line that demonstrates many features of primary RPE cells, and therefore they can serve as a valuable tool in RPE research. This cell line should provide an excellent model for the study of RPE cells in culture and will allow more extensive experiments to be performed on retinal diseases. From the transformation tests done, the tRPE cells do not show major transformation characteristics nor obvious changes to the cells phenotype, although further investigation is required to ensure that no genetic mutations have spontaneously occurred that might alter the cells genotype. Also, the tRPE cells have the ability to incorporate lipofuscin granules and the bis-retinoid A2E, which currently are being investigated further.

## Methods

### Isolation and culture of primary RPE cells

RPE cells were isolated by a modified technique described by Chang et al. [81]. Briefly, primary RPE cells were obtained from fresh calf eyes (Brown Packaging, S. Holland, IL). Extraneous fat and muscle was removed from the periphery of the eyeballs. After soaking in 70% ethanol for 30 seconds, the sclera was cut mid-coronal and the anterior portion of the eye was removed. Cold 4°C phosphate buffered saline (PBS) was pipetted into the eyecup with care so that the level of the PBS was below the edge of the incision, so as to not contaminate the eyecup with choroidal melanocytes, and the neural retina was allowed to float off. The optic nerve was cut, and the neural retina was removed. The eyecup was rinsed with cold PBS and then bathed in 0.25% trypsin-EDTA (Sigma-Aldrich; Milwaukee, WI) for 10 minutes at 37°C. The eyecup was brushed gently to remove the cells. They were plated in multi-well tissue culture dishes and grown at 37°C and 2% CO_2 _until confluent. The cultures were re-fed every three days with DMEM (Sigma-Aldrich; Milwaukee, WI) plus 10% FBS (Invitrogen, Carlsbad, CA) and penicillin/streptomycin (Sigma-Aldrich; Milwaukee, WI).

### Clone isolation

Originally, seven different primary explants were observed, and they were removed using cloning rings. Each of these clones was grown under low-density conditions, and a single colony was isolated from each. The cells were passaged by trypsinization when confluent, approximately every 2–3 days, and they were split at a 1:2 ratio. Of the seven original clones, only one surpassed the 50-passage mark and was designated tRPE.

### Isolation of primary RPE sheets

RPE sheets were isolated by a modified technique described by Ho [82]. Briefly, 100 μL of 12% 175 Bloom porcine gelatin (Sigma-Aldrich; Milwaukee, WI) was layered over RPE cells from a chilled eyecup. The eyecup was placed in a 4°C cold room for 10 minutes to allow the gelatin to solidify. The gelatin, along with the RPE layer, was removed with sterile forceps and placed on glass microscope slides in culture dishes and incubated for 5 minutes to melt the gelatin. The culture dishes were rinsed with PBS and photographed.

### Exogenous melanin endocytosis

Melanosomes were isolated from RPE cells from calf eyes by a technique described by Feeney-Burns [83]. Briefly, RPE cells were extracted from bovine eyes. Nitrogen cavitation was used to lyse the cells. Using a 2.2 M and 1.38 M sucrose density gradient, the cell suspension was separated. The test tubes were centrifuged at 10,000 rpm for 60 minutes. The upper two layers were removed and the melanosomes were lyophilized and frozen until needed. Prior to feeding the cells, the melanosomes were washed once with 70% ethanol to sterilize the solution and then centrifuged. The supernatant was removed, and the melanosomes were resuspended and centrifuged in PBS three times to remove excess sucrose. The melanosomes were added to the media of confluent tRPE cells as described by Boulton and Marshall [53] for every 3 days for two weeks.

### RT-PCR

Total RNA was extracted from primary RPE cells, tRPE cells grown on plastic for 3 months, tRPE cells grown on bovine corneal epithelium derived extra cellular matrix and supplemented with 1 ng/ml bFGF, 50 ng/ml all-trans retinoic acid and 0.25 mM ascorbic acid (Sigma-Aldrich; Milwaukee, WI) using guanidine HCl/Cesium Chloride Ultracentrifugation [84]. CHO AA8 cells (ATCC) were utilized for negative controls for RPE-specific genes. A phenol/chloroform purification followed. The mRNA was isolated using Oligotex^®^, RT-PCR was performed using Sensiscript™ RT Kit and *Taq *PCR Core Kit all from Qiagen (Valencia, Ca) according to manufactures protocol. The primers used in this study have been included in Table 1.

**Table 1:** Primers used for the reverse transcriptase PCR

Gene		Primer Sequence	Length	Tm (oC)	Product size
CRALBP	forward	AGA ACT CTC CAG CTT CTA CCA GGA	24 bp	60.2	301 bp
	reverse	AGC TTG GGA GGA GGA TGA CAT CTG	24 bp	59.8	

β-Actin	forward	TCA TGA TCG AGA CCT TCA ACA CCC	24 bp	60.3	288 bp
	reverse	AGC AGA GCT TCT CCT TGA TGT CAC	24 bp	60.1	

RPE-65	forward	CCA GGG CTG CTG AGG TGG TTA ACA	24 bp	60.0	278 bp
	reverse	GTG CCT GTC TCA CGA AGT ACG ATT	24 bp	60.1	

Vimentin	forward	ATG AAG GAA GAG ATG GCT CGT CAC	24 bp	59.9	173 bp
	reverse	TCC AGA TTG GTT TCC CTC AGG TTC	24 bp	60.0	

Primer sequences, lengths, melting temperatures and product sizes have been included used in the identification of RPE-specific genes and control genes for primary RPE cells and tRPE cell cultures.

The CRALBP primer was determined by using the bovine cDNA sequence [GenBank; J04214], which spans 867 to 1168 base pairs. This is homologous to exon 8 of the human CRALBP gene sequence [GenBank, gi:598228]. The β-actin primer was determined by using the bovine cDNA sequence [GenBank, J04214], which spans 404 to 692 base pairs. This is homologous to exon 3 of the human β-actin gene sequence [GenBank, gi:177967]. The RPE65 primer was determined by using the bovine cDNA sequence [GenBank, L11356], which spans 1592 to 1870 base pairs. This is homologous to exon 4 of the human RPE-65 gene sequence [GenBank, gi:67188783]. Finally, the vimentin primer was determined by using the bovine mRNA sequence [GenBank, L13263], which spans 1114 to 1286 base pairs. This is homologous to exons 6 and 7 of the human vimentin gene sequence [GenBank, gi:340230].

### Immunocytochemistry

To determine whether the mRNA was translated into proteins, immunocytochemistry was performed. This was done to ensure that the mRNA was not degraded and it produced sufficient protein to show that the cells were similar to the commonly used cell line, ARPE-19. Passage 102 tRPE cells were grown on bovine corneal epithelium derived extra cellular matrix in 6-well plates and supplemented with 1 ng/ml bFGF, 50 ng/ml all-trans retinoic acid and 0.25 mM ascorbic acid (Sigma-Aldrich; Milwaukee, WI) to differentiate them. Control tRPE cells were used that had not been incubated with primary antibodies.

### Zonula Occludens-1

Confluent tRPE cells were allowed to remain attached to the extracellular matrix for eight weeks to ensure confluency and differentiation. The media was removed and the cultures were washed two times with PBS. The cells were fixed in 4% paraformaldehyde at room temperature for 10 minutes and washed in PBS three times at 10 minutes each.

The monolayer was blocked with 3% Fraction V BSA (Sigma-Aldrich; Milwaukee, WI) in TTBS (250 mM NaCl, 0.05% Tween 20 at a pH of 7.61) for 1 hour at room temperature on an orbital shaker. Goat polyclonal anti-ZO-1 (Santa Cruz Biotechnology; Santa Cruz, California) was used at a dilution of 1:100 in blocking buffer. The primary antibody was incubated overnight at 4°C while shaken. The culture was washed two times in TTBS for 10 minutes each prior to incubation with the secondary antibody. The washing between antibody incubation was necessary to reduce the background.

The cells were then incubated and shaken at room temperature for one hour with a fluorescein-isothiocyanate (FITC) conjugated bovine anti-goat IgG (Santa Cruz Biotechnology; Santa Cruz, California) diluted to 1:500 in blocking buffer. The monolayer was washed three times in TTBS at room temperature for 5 minutes each and then covered in 1 ml of PBS. The nuclei were counterstained with Hoechst 33258 at a final concentration of 1 mg/ml and allowed to incubate for 15 minutes at 37°C.

### RPE65

A 4% paraformaldehyde solution was freshly prepared in PBS. Cultures of differentiated tRPE cells were fixed for 30 minutes at 4°C. They were then washed in cold TTBS with 0.1% Triton X-100 three times at 10 minutes each on an orbital shaker. The cells were blocked in 3% BSA in TTBS for 1 hour while shaken. They were then incubated overnight while shaken at 4°C with a 1:40 dilution of monoclonal rabbit anti RPE65 which was kindly donated by Dr. Rosalie Crouch from the Department of Ophthalmology at the Medical University of South Carolina. The cells were washed three times at 10 minutes each in TTBS and then incubated with FITC-conjugated polyclonal donkey anti-rabbit IgG (Santa Cruz Biotechnology; Santa Cruz, California) at room temperature at a final dilution of 1:200. They were then washed three times in TTBS for 10 minutes each and then washed twice with PBS and finally 1 mL of PBS was added. Nuclei were counter stained with Hoechst 33258 for 15 minutes at 37°C.

### Vimentin

Sub-confluent tRPE cells were used to show better spreading of the cytoskeleton. The media was removed and the culture was twice washed with PBS and removed. Freezing them for two minutes to break open the cell wall permeabilized the cells. The culture was fixed with -20°C methanol for 10 minutes and then washed three times with PBS for 5 minutes each washing. Blocking was done with 3% BSA for one hour and then incubated overnight at 4°C with Rabbit anti-vimentin (Santa Cruz Biotechnology; Santa Cruz, California) used at a dilution of 1:100. The cultures were washed twice with TTBS before adding FITC-conjugated polyclonal donkey anti-rabbit antibody (Santa Cruz Biotechnology; Santa Cruz, California) at a 1:200 dilution in 1% BSA and allowed to incubate for 1.5 hours. The culture was washed and the nuclei were stained in the same manner as the ZO-1 inoculation.

### Pan-Cytokeratin

Confluent tRPE cells were permeabilized, fixed and blocked in the same manner as the vimentin immunocytochemistry culture. Rabbit anti pan-cytokeratin (Santa Cruz Biotechnology; Santa Cruz, California) at a dilution of 1:200 was incubated at 4°C overnight. The vimentin protocol was followed for the remainder of the experiment.

### Bestrophin

The cultures were fixed in 4% paraformaldehyde in a 0.1 M phosphate buffer at a pH of 7.2 for 20 minutes at room temperature. The cells were washed three times in PBS with 0.1% Triton X-100 for a total of 30 minutes on a shaker. The cells were blocked with 1% BSA for one hour and washed twice with TTBS. Polyclonal rabbit anti-bestrophin, which was kindly donated by Dr. Alan Marmorstein from the Cole Eye Institute, was used at a dilution of 1:250 at 4°C overnight. The cells were washed four times with TTBS for five minutes each wash. For the remaining steps, the vimentin protocol was followed.

### Imaging

All cells were viewed with a Nikon Eclipse TS100 inverted microscope with fluorescence attachments. An OSRAM HBO Mercury Short Arc lamp was used to illuminate the cells with the proper filter cubes in place. Four filter cubes were used. The CFP/dsR FRET filter cube had an excitation range of 426–446 nm and an emission range of 460–500 nm with a dichromatic mirror cutoff at 455 nm. The UV-2E/C filter cube had an excitation range of 340–380 nm and an emission range of 435–485 nm with a dichroic mirror cutoff at 400 nm. The TRITC HQ filter cube had an excitation range of 530–550 nm and an emission range of 590–650 nm with a dichromatic mirror cutoff at 565 nm. The FITC HQ filter cube had an excitation range of 450–500 nm and an emission range of 510–560 nm with a dichromatic mirror cutoff at 505 nm. The cells were imaged using a Nikon Cool-Pix camera at 3.5 megapixels attached to the microscope. Images were processed using Adobe Photoshop.

### Extra-cellular matrix preparation

Extra-cellular matrix (ECM) preparation using primary bovine corneal endothelial cells was performed by a technique described by Campochiaro and Hackett [24]. Briefly, the cornea was removed from the eye after soaking the eyeball in 70% ethanol for 30 seconds. The cornea was removed and the interior of the cornea was trypsinized for 5 minutes and the resulting corneal endothelial cells were plated on tissue culture plates in DMEM supplemented with 20% FBS (Invitrogen, Carlsbad, CA), 1 ng/ml bFGF, 50 μg/ml endothelial cell growth supplement, 100 μg/ml heparin sulfate and penicillin/streptomycin (Sigma-Aldrich; Milwaukee, WI). They were allowed to grow to confluency and passaged no more than twice before the final plating. On the final plating, the cells were fed every three days for two weeks and then the media was changed to DMEM plus 10% FBS and penicillin/streptomycin for the final two weeks.

The ECM was harvested by rinsing the cells with cold PBS and incubating them with a 0.5% Triton-X 100 and 20 mM ammonium hydroxide solution for 8–10 minutes at 37°C. The ECM was then rinsed 4 times with cold PBS and the tissue culture plates were stored in a 4°C refrigerator with PBS until needed with the ECM covered in PBS.

### Chromosome preparation

Standard procedures were used to obtain chromosome preparations. Specifically, 0.8% Sodium Citrate was used as a hypotonic solution and a 3:1 methanol/acetic acid solution was used to fix the cells with the cells incubating at 4°C in fixative for 1 hour prior to spreading [85]. Staining was done using Carrazi's Alum Hematoxylin and was viewed under a Zeiss microscope with an attached video camera. The chromosomes were scored using a CRT screen to amplify the image of each cell.

### Suspension growth

Approximately 3.8 × 10^5 ^tRPE cells and CHO-AA8 cells were incubated in 15 ml of DMEM plus 10% FBS and penicillin/streptomycin using a roller drum apparatus. The cells were counted with a Coulter cell counter on days 1,5,7,9 and 11.

### ^3^H emulsion autoradiography

Autoradiography was performed *in situ *by a modified technique described by Hopwood and Tolmach [86]. In 35 mm^2 ^tissue culturing plates, 50,000 tRPE cells were added and allowed to attach overnight. The cells were grown to confluency in DMEM plus 10% FBS over the next 15 days. The plates were incubated with ^3^H thymidine (ICN Radiochemicals) for 4 hours at 37°C at a final concentration of 0.93 μCu/mL. After incubation, pulse termination was performed by aspirating off the media.

For processing, the cells were washed twice with cold PBS, followed by a 4% perchloric acid solution incubation at 4°C for 4 days. The cells were fixed for 10 minutes in 3:1 methanol/acetic acid and allowed to dry overnight at 4°C, and were overlayed with NTB-3 (Kodak) autoradiographic emulsion diluted 1:1 with ddH_2_O in a dark room, which was then poured into the plates, and allowed to solidify. The plates were placed in light-tight boxes wrapped in foil, and placed at 4°C for five days.

The plates were developed by pouring 20°C warmed KODAK D-19 developer in the wells, were rinsed with warmed ddH_2_O and then fixed with KODAK Fixer for 3 minutes. The plates were finally rinsed carefully with room temperature ddH_2_O. The plates were then soaked in Carrazi's Alum Hematoxylin (0.1% hematoxylin, 0.21 M aluminum ammonium sulphate, 20% glycerol, and 1.1 mM Sodium iodate) for 15 minutes and then were rinsed. They were allowed to dry and harden for 1 week. The bottom of each well was punched out using a homemade well cutter. Each plate was coded to ensure that the experimenter had no bias. The number of heavily labeled cells for each coded plate was determined by observation using a 2 × 2 cm^2 ^grid (produced in-house) that was subdivided into 25 4 × 4 mm^2 ^zones.

## Authors' contributions

TEL drafted the manuscript, performed the experiments and designed the protocol. TDG and ERG designed the protocol and coordinated the study. All authors have read and approved the final manuscript.
